# Association between US Pharmacopeia (USP) monograph standards, generic entry and prescription drug costs

**DOI:** 10.1371/journal.pone.0225109

**Published:** 2019-11-12

**Authors:** Irene B. Murimi-Worstell, Jeromie M. Ballreich, Marissa J. Seamans, G. Caleb Alexander

**Affiliations:** 1 Department of Epidemiology, Johns Hopkins Bloomberg School of Public Health, Baltimore, Maryland, United States of America; 2 Center for Drug Safety and Effectiveness, Johns Hopkins University, Baltimore, Maryland, United States of America; 3 Department of Health Policy & Management, Johns Hopkins Bloomberg School of Public Health, Baltimore, Maryland, United States of America; 4 Department of Mental Health, Johns Hopkins Bloomberg School of Public Health, Baltimore, Maryland, United States of America; 5 Division of General Internal Medicine, Department of Medicine, Johns Hopkins Medicine, Baltimore, Maryland, United States of America; University of California Berkeley, UNITED STATES

## Abstract

Despite the importance of pharmacopeial standards, little is known regarding their effect on drug competition. Such information is of particular relevance given the rising costs of prescription drugs and the focus of policy-makers and other stakeholders on addressing these costs. We examined 982 prescription drugs approved by U.S Food and Drug Administration since 1982 to examine the association between U.S. Pharmacopeia (USP) standards, generic entry and prescription costs. The presence of a USP drug product monograph was not associated with the time to the third generic entrant or with the likelihood of having a generic competitor. However, on average, drugs with USP drug product monographs had approximately fifty percent more generic manufacturers in the U.S. than their counterparts after accounting for factors such as market volume, age, route of administration and vintage. This greater competition was associated with an approximate savings of $6.22 billion in 2016, suggesting that USP drug product monographs may play an important role in promoting pharmaceutical competition and reducing prescription drug costs.

## Introduction

Rapidly rising prescription drug costs in the United States represent a significant challenge to patients, providers and the health care system alike. [1] For example, among patients currently taking a prescription medication, 19% report that they or a family member have cut pills in half or skipped doses while 24% report that they or a family member have failed to fill a prescription because of cost.[2]

One important driver of increased pharmaceutical costs is the use of single-source drugs, which among the commercially insured represent only 10% of prescriptions filled yet account for 63% of pharmaceutical spending.[3] Many single-source drugs are protected by patents or exclusivity provisions that limit new competitors to the market. However, others represent products where generic competition could create competition and lower pharmaceutical costs. For example, in the 10-year period ending in the 2018, generics saved the U.S healthcare system an estimated $2 trillion dollars. [4]

Given the potential cost-savings associated with greater generic competition, this has been a focus of both regulators and policy-makers. For example, the 2012 Generic Drug User Fee Amendments provided resources for speeding the approval of safe and effective generic drugs through the Abbreviated New Drug Application (ANDA) mechanism. [5] Similarly, U.S Food and Drug Administration (FDA) has disseminated industry guidelines to promote efficient ANDA submissions and approvals. [6]

The U.S. Pharmacopeial Convention (USP) develops monographs and physical standards for approved pharmaceutical products [7–8], and is vested in assuring that patients have access to quality prescription drugs. USP monographs include the name of the ingredient or preparation; the definition; labeling, packaging, storage requirements; and the specifications, which consist of a series of tests, procedures for the tests, and acceptance criteria. These tests and procedures often require the use of official USP physical reference standards. Pharmaceutical products will have the required strength, quality, and purity if they conform to the requirements of the monograph and applicable general chapters in the United States Pharmacopeia and the National Formulary (USP-NF).[9] The development of a public standard is dependent on factors including willingness of industry partners to participate in the process and complexity of the product or approval pathway. Majority of monographs are developed using information provided by manufacturers while others are developed internally by USP without manufacturer support. All standards irrespective of industry support are revised and approved by independent scientific experts, with support from USP staff, through a public process. [7–13]

Several studies have suggested that USP standards aid in drug development and in quality assurance. [10, 13] For example, an excipient in a new drug product that meets USP monograph standards can cite the standard in regulatory filings without further characterization thus potentially decreasing the time and cost of product development. [13] Despite these reported benefits to the drug development process, to our knowledge, the association between these monographs and the entry of additional generic manufacturers to the market and, by extension, the effect of the monographs on drug costs, has not been quantified. Thus, we performed a comprehensive assessment of 982 prescription drugs approved by FDA since 1982 to quantify the association between USP standards, generic market entry and prescription expenditures.

There are two main types of monographs. We focused primarily on USP drug product monographs, which outline the identity, strength, quality and purity of a specific product, rather than substance monographs, which describe the drug substance but not the nature of the finished form. We reasoned that the product-specific guidance included in drug product monographs included information that is more relevant to potential generic entrants.

## Study data and methods

### Data

We used four data sources to conduct our analysis. First, we used the electronic version of the FDA Orange Book to derive information on all drugs approved in the United States. [14] The Orange Book includes ingredient names, route of administration, the date and type of the drug application, and the name of the applicant. Second, we linked each product within the Orange Book to the USP Monograph Directory. [15] This linkage, which was performed at an ingredient-route level (e.g., furosemide–intravenous formulation), allowed for us to assess, for each ingredient-route combination in the Orange Book, whether and on what date a USP monograph had been established. Third, we used the IQVIA World Review Track Data (1997–2016) to obtain sales data both in dollar and prescription volume for the U.S drug market at the molecule and formulation level.[16] Finally, we used manual matching by drug product name and the electronic version of the FDA’s National Drug Codes (NDC) directory to link information derived from the Orange Book with information provided by IQVIA [17].

### Study sample

**Fig 1:**
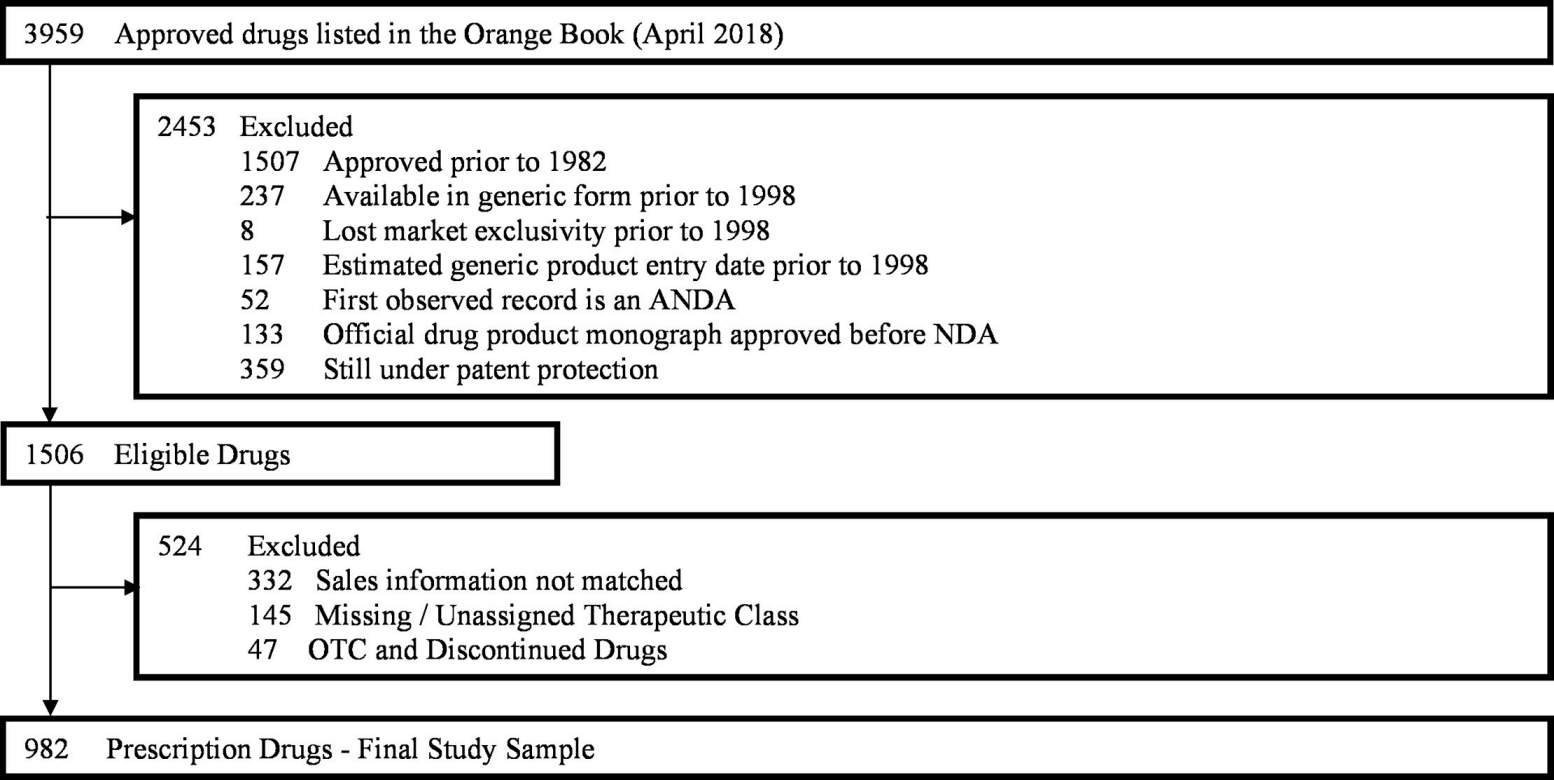
Study sample derivation. ANDA = Abbreviated New Drug Application; NDA = New Drug Application; OTC = Over-the-counter.

We identified 3959 products in the FDA Orange Book that had been approved prior to April 3, 2018. Each distinct ingredient-route combination was analyzed separately, and no biologic products were included in our analysis. Out of these 3959 products, we excluded 1552 that were first approved prior to 1982 since the FDA Orange Book does not list the precise date of approval for applications approved before 1982. The approval dates were needed to determine the age of the drug and the order in which different manufacturers of any given drug entered the market. Given our interest in evaluating the product’s market size right before generic market entry or loss of exclusivity, we also excluded 402 drugs that had generic alternatives or had lost market exclusivity prior to 1998, since we did not have IQVIA sales data regarding these products. We excluded another 133 products with a drug product monograph that became official before the New Drug Application (NDA), 359 that were still under patent protection, 332 that could not be matched to the IQVIA data, 145 that could not be assigned to a therapeutic class and 47 over-the-counter (OTC) or discontinued drugs. Thus, our final sample consists of 982 prescription products (**Fig 1**).

### Key measures

We used three different measures to examine the effect of USP monographs on generic competition. First, we characterized the effect of monographs on the availability of at least one generic manufacturer. In other words, we sought to answer the question “Are products with USP monographs more likely to have a generic entrant than their counterparts”? Second, we quantified the impact of monographs on the time between the second and third generic manufacturer’s entry into the market. In other words, we sought to examine whether, among products with two generic competitors, the presence of a monograph speeds the entry of the third generic entrant to market. We focused on the third, rather than earlier entrants because litigation and the 180-day generic drug exclusivity provision may delay marketing of the first and second generic respectively. Third, we examined the effect of monographs on the number of generic manufacturers ultimately entering the marketplace.

### Statistical analysis–effect on generic entry

First, we used descriptive statistics to characterize the prevalence and timing of USP drug product monograph standards relative to the market entry of subsequent manufacturers. Second, we used generalized estimating equations with robust standard errors to assess the association between having a USP drug product monograph with the availability of at least one generic manufacturer. Next, limiting the analysis to drugs that had two or more generics, we used Cox proportional hazards models to evaluate the impact of drug product monographs on the time between when the second and third generic manufacturer’s applications were approved. Drugs were followed until the outcome of interest occurred namely the approval of the third generic manufacturer or until the end of the study on April 3, 2018. Finally, in our main analytical model, we used generalized estimating equations with robust standard errors to estimate the effect of having a drug product monograph before the first generic entered the market on the total number of generic manufacturers ultimately entering the market.

We accounted for a variety of additional factors that might impact generic entry in our models, whether by influencing a manufacturer’s decision to donate a drug product monograph or by directly impacting the likelihood of generic entry by a firm. These factors included: (1) the availability of a USP drug substance monograph; (2) the market size of the drug of interest, which was approximated using the total sales for the product in the year before the first generic entered the market or when the drug lost exclusivity; (3) the age of the drug which was defined as the number of years since the drug’s NDA was approved; (4) the drug vintage, which was defined as the order in which the product was approved relative to other drugs in its class; and (5) the route of administration. In addition, each of the models accounted for the clustering effect within drugs of the same therapeutic class.

### Statistical analysis–effect on drug expenditures

Using the output from the main statistical model which examined the effect of drug product monographs on the number of generic entrants, we estimated the cost savings associated with USP drug product monographs by comparing actual sales for a drug with a drug product monograph for a given year with the predicted sales for the same drug if no drug product monographs were established. The number of manufacturers for a drug were based on the number of approved generic manufacturers within the unique ingredient route combination. We then predicted the number of manufacturers if no USP monographs were established using the results from earlier regression analysis.

We compared the predicted and actual number of manufacturers, and using a recent analysis on generic competition and price [18], we estimated new prices based on relative difference in number of manufacturers. We multiplied the estimated new prices by actual sales volume to derive the predicted sales under the counterfactual, which is what would have happened if no USP drug product monographs existed. The difference between predicted and actual sales estimated the cost savings associated with USP monographs Eqs 1–4 summarize the computations outlined above.

$$\begin{array}{c} N_{P,i}=\frac{N_{A,i}}{IRR}\\ N_{P,i}=Number\;of\;predicted\;generic\;manufacturers\;for\;drug\;i\\ N_{A,i}=Number\;of\;actual\;generic\;manufacturers\;for\;drug\;i\\ IRR:Incidence\;rate\;ratio \end{array}$$ Eq 1

$$\begin{array}{c} P_{P,i}=\frac{D_{P}}{D_{A}}*P_{A,i}\\ P_{P,i}=Predicted\;price\;for\;drug\;i\\ P_{A,i}=Actual\;price\;for\;drug\;i\\ D_{P}=Predicted\;price\;reducdtion\;based\;on\;number\;of\;predicted\;generics\\ D_{A}=Actual\;price\;reducdtion\;based\;on\;number\;of\;actual\;generics \end{array}$$ Eq 2

$$\begin{array}{c} S_{i}=P_{P,i}*V_{A,i}\\ S_{i}=Predicted\;savings\;for\;drug\;i\\ V_{A,i}=Actual\;volume\;for\;drug\;i \end{array}$$ Eq 3

$$\begin{array}{c} S_{t}=\sum S_{i}\\ S_{t}\;Total\;Savings\;for\;year\;t\;is\;sum\;of\;individual\;drug\;savings\;for\;year\;t \end{array}$$ Eq 4

### Sensitivity analyses

We performed a series of sensitivity analyses to assess the robustness of our findings. First, given that the timing of the drug product monograph is likely to be associated with its impact on the level of generic competition, we examined the effect of establishing the drug product monograph before the second and before the third generic manufacturer on the total number of manufacturers for the drug. Second, given the notable differences in the sizes of the drug classes, we evaluated the effect therapeutic class size on the estimated effect of the drug product monograph by repeating the main analysis using drug classes of varying sizes. Third, we observed significant variation in the time between NDA approval and the first generic manufacturer’s entry to the market across the drugs. This variability could potentially influence the probability of drug product monograph donation and the monograph’s impact of generic market. To examine the impact of this variation on the estimated effect of drug product monographs, we reanalyzed the main model excluding drugs with short intervals between the NDA and first generic approval. Fourth, since the likelihood of generic entry might vary based on the pharmacologic complexity of a given product, we repeated the main model after stratifying products based on the route of administration. Lastly, since not all first generic manufacturers have 180-days of market exclusivity and given the notable price reduction associated with the entry of the second generic manufacturer into the market, we performed a secondary analysis examining the association having a drug product USP monograph the time between the first and second generic entrant.

Analyses were performed using SAS, version 9.4 (SAS Institute Inc) and Stata, version 13 (StataCorp, College Station, Texas). Two sided p values of less than 0.05 were considered statistically significant. The study was exempted from a Johns Hopkins Bloomberg School of Public Health Institutional Review Board review.

## Results

### Prevalence and timing of official USP monograph standards

Approximately two in five (n = 396, 40.3%) of the 982 prescription drugs analyzed had a drug product monograph and three quarters (n = 735, 74.9%) had a drug substance monograph. Orally administered drugs had the highest prevalence of both drug product and drug substance monographs among all products. (**Table 1**) Half (51%) of the drug product monograph standards were established before the first generic entered the market.

**Table 1:** Prevalence of official drug product and drug substance monographs, stratified by route of administration.

Route, N (%)	Total	*with* Drug Product Monographs	*with* Drug Substance Monographs
Inhalation	31	6 (19.4)	19 (61.3)
Intravenous	183	66 (36.1)	109 (59.6)
Oral	553	269 (48.6)	439 (79.4)
Topical	93	29 (31.2)	73 (78.5)
Other	122	26 (21.3)	95 (77.9)
Total	982	396 (40.3)	735 (74.9)

### Monograph association with presence of at least one generic entrant

In unadjusted analysis, products with drug product monographs were more likely to have at least one generic manufacturer (91% vs 45%, odds Ratio [OR] 3.01, 95% confidence intervals [CI] 2.07–4.38) **(Table 2)**. However, after adjusting for potential confounders, there was no statistically significant difference in the likelihood of having a generic manufacturer based on the presence of a USP drug product monograph (adjusted OR 0.64, CI 0.38–1.09).

**Table 2:** Association between official drug product monograph and generic competition*.

Association Between Official Drug Product Monograph and…	Effect Estimate (95% confidence intervals)
…availability of generics	Crude OR: 3.01 (2.07, 4.38)
	Adj. OR: 0.64 (0.38, 1.09)
…time between approval of second and third generic manufacturer	Crude HR: 0.88 (0.73, 1.08)
	Adj. HR: 0.99 (0.80, 1.24)
…number of generic manufacturers (Main Analysis)	Crude IRR: 0.01 (0.00, 0.01)
	**Adj. IRR: 1.53 (1.15, 2.03)**

Abbreviations: Adj.: Adjusted, HR: Hazard Ratio, IRR: Incidence Rate Ratio, OR: Odds Ratio

*Adjusted models accounted for presence of a drug substance monograph, age of the drug, market size of drug and drug vintage, route of administration and correlation between drugs of the same class

### Monograph association with time to third generic entrant

**Fig 2:**
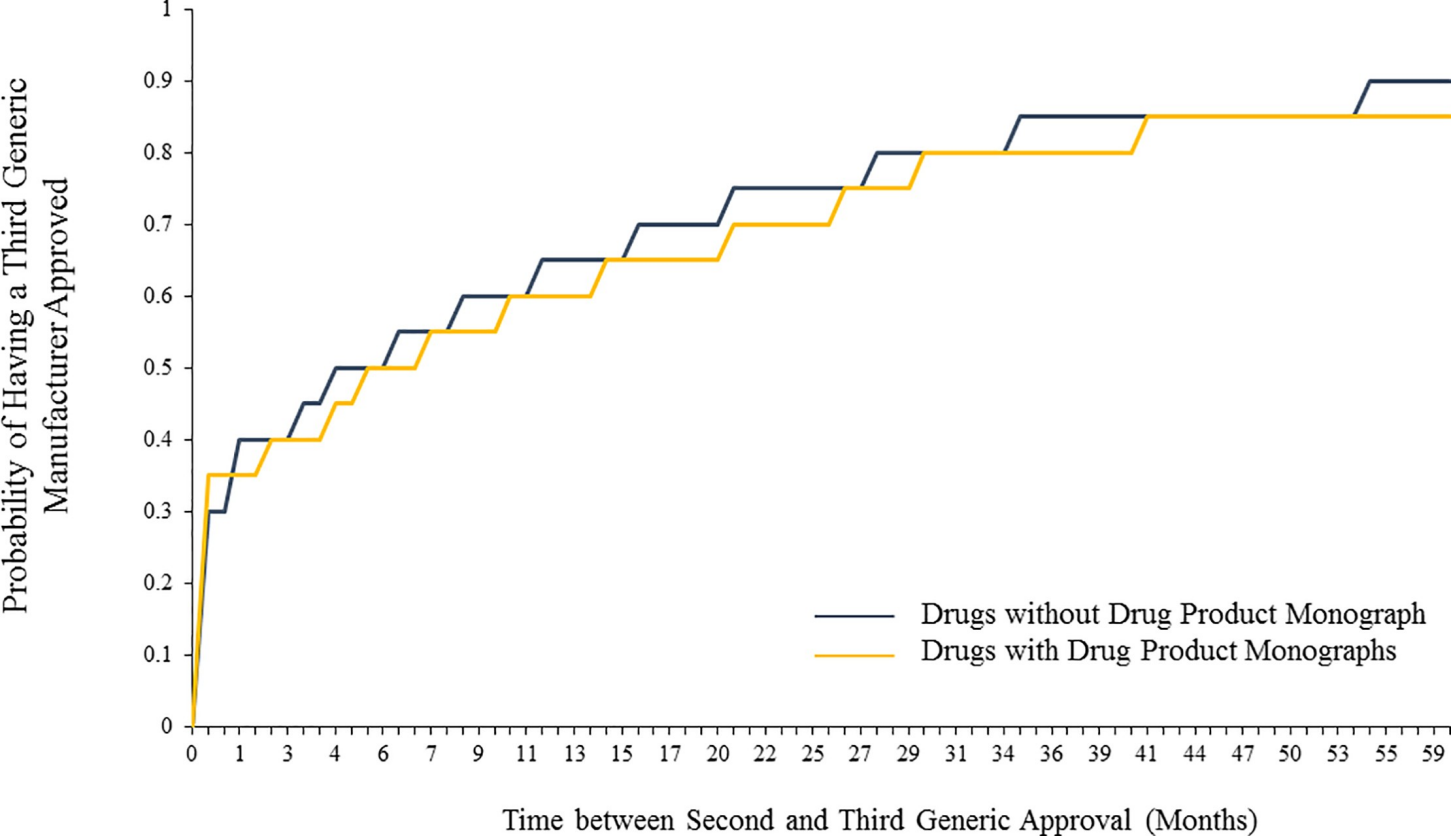
Probability of having a third generic manufacturer approved, stratified by availability of an official drug product monograph.

Of the 508 drugs that had two or more generic manufacturers, 166 (32.6%) had drug product monographs that were established prior to the second generic. On crude analysis, the median time between the second and the third generic manufacturer’s approval was 180 (CI 103–287) days and 163 (CI 98–212) days for drugs with and without drug product monographs respectively. (**Fig 2**) After adjusting for potentially confounding covariates, there was no statistically significant association between the presence of a drug product monograph and the timing of the third generic entrant (adjusted hazard ratio [HR] 0.99, CI 0.80–1.24).

### Monograph association with number of generic entrants

On average, drugs with drug product monographs had 7.2 generic manufacturers per drug compared to the 1.7 for those without published standards. After accounting for relevant confounders, drugs that had a published drug product monograph prior to the first generic entrant had 1.53 (95% CI: 1.15–2.03) times the number of generic alternatives as those that never had issuance of a drug product monograph. In secondary analysis, drugs that had a drug product monograph before the second generic entrant had 1.24 times (95% CI: 1.03–1.49) the number of generic manufacturers, while those whose drug product monographs were established prior to the third generic the rate of generic market entry was 1.19 (95% CI: 1.00–1.42) times higher than for those who never had a drug product monograph.

### Cost savings associated with USP monographs

Cost savings associated with USP drug product monograph issuance was estimated to be $5.09 billion and $6.22 billion annually in 2015 and 2016 respectively. These cost savings were driven by our estimated 386 and 417 additional generic manufacturers on the market due to USP monographs. These additional manufacturers represented 28% of the total number of generic manufacturers in our study sample.

### Sensitivity analyses

Results of sensitivity analyses were largely consistent with our main findings. For example, there were no substantive differences in the estimated effect of USP drug product monographs on the number of generics when we restricted the analysis to drug classes with fewer drugs. Similarly, the reanalysis of the main model using different thresholds to exclude drugs classes of various sizes or to exclude products that have short durations between the date of the NDA and the date of the first generic yielded substantively similar results. However, after stratification by different routes of administration, the effects of monographs were statistically significant only when examining orally administered products. **(Table 3 and Table 4)**

**Table 3:** Sensitivity analyses examining association between official drug product monograph and number of generic manufacturers*.

Description of the Study Population	IRR (95% CI)
Secondary Analysis: Varying the Timing of the Drug Product monograph	
Drug monograph issued before 2^nd^ generic entry	1.24(1.03, 1.49)
Drug monograph issued before 3^rd^ generic entry	1.19(1.00, 1.42)
Sensitivity Analysis 1: Effect of the Time between NDA and First Generic Approval	
First generic approved >3 years after NDA	1.60 (1.20, 2.14)
First generic approved >5 years after NDA	1.60 (1.18, 2.16)
First generic approved >8 years after NDA	1.85 (1.30, 2.63)
Sensitivity Analysis 2: Effect of Therapeutic Drug Class Size	
Therapeutic classes with <10 drugs	1.54 (0.86, 2.76)
Therapeutic classes with <21 drugs	1.65 (1.11, 2.43)
Therapeutic classes with <54 drugs	1.66 (1.23, 2.24)
Sensitivity Analysis 3: Route-Specific Effects	
Orally administered drugs	1.80 (1.34, 2.43)
Injectable drugs	1.11 (0.55, 2.25)
Topically applied drugs	0.88 (0.51, 1.53)
Other routes of administration	1.26 (0.60, 2.69)

Abbreviations: IRR: Incidence Rate Ratio, CI: Confidence Interval

*: All models account for the presence of a drug substance monograph, age of the drug, market size of drug and drug vintage, route of administration and correlation between drugs of the same class.

**Table 4:** Secondary analyses examining association between official drug product monograph and time between the approval of the first and second generic.

Association Between Official Drug Product Monograph and…	Effect Estimate (95% confidence intervals)
…time between approval of first and second generic manufacturer*	Crude HR: 0.82(0.78, 1.01)
	Adj. HR: 0.88 (0.66, 1.16)

*Adjusted model accounts for the presence of a drug substance monograph, age of the drug, market size of drug and drug vintage, route of administration and correlation between drugs of the same class.

## Discussion

Despite the importance of pharmaceutical standards such as those established by the U.S. Pharmacopeia (USP), little is known regarding their effect on drug competition. Such information is of particular relevance given the rising costs of prescription drugs and the focus of policy-makers and other stakeholders on addressing these costs. We analyzed 982 products approved by the U.S. FDA since 1982. After accounting for factors such as the drug volume, age, vintage and route of administration, on average, drugs with USP drug product monographs had approximately fifty percent more generic manufacturers than their counterparts. We estimate that the greater generic competition ensuing from these entrants was associated with an approximate savings of $6.22 billion in 2016. These results are important because of the heightened concern among patients, providers and policy-makers regarding the high cost of prescription drugs, and the intense interest of these and other parties in identifying methods to promote more robust marketplace competition.

While monographs were associated with a greater number of generic entrants, interestingly, we did not find an association between monographs and the presence of any generic entrant. These results suggest that the factors driving the first generic entry into the market differ from those influencing subsequent manufacturers’ decisions. One of these factors may be the 180-day period of exclusivity given to the first generic entrant which provides a financial incentive to manufacturers to enter the market even in the absence of monograph standards.[19] On the other hand, USP monograph standards may serve to incentivize subsequent generic manufacturers by providing ready access to validated manufacturing procedures and product standards.[10] While our analysis did not find an association between drug product monographs and the speed of generic entry, the availability of these widely-accepted monographs may represent a resource savings to manufacturers by reducing or eliminating the need to develop in-house testing procedures.[13] Interestingly, the association between drug product monographs and generic competition was only discernible for orally administered products.

Our findings suggest USP drug product monographs may be an important facilitator of generic competition. However, there are other important barriers to such competition. For example, strategic delay tactics such as reverse payment patent settlements, through which the innovator pays the generic manufacturer not to market their approved product, have become increasingly common and serve to impede the cost-saving function of generic competition. [20–21] A report by the Federal Trade Commission, estimates that these pay to delay tactics costs consumers and payers upwards of $3.5 billion annually. [22] In addition, restricted access to samples of originator product through exclusionary risk evaluation and management strategies (REMS) may impede generic manufacturers’ ability to duplicate single source drugs [23–25] and are a focus of the CREATES ACT of 2017.[25, 26] The availability of monographs is not the single answer to the anti-competitive practices in the pharmaceutical market place. However, by providing publicly available product standards against which manufacturers can measure their products, monographs level the playing field and could be part of the multifaceted solutions needed to promote a more robust generic market.

Our study had several limitations. First, it is possible that unmeasured or unmeasurable factors (e.g., reverse payment patent settlement agreements)confound the primary association of interest. However, we adjusted for many important determinants of market entry, and we performed several sensitivity analyses that suggested our results were robust to different assumptions. Second, since the USP relies upon manufacturer donations for drug product monograph development, reverse causation is theoretically possible, since drugs with multiple manufacturers have more potential drug product monograph donors. To minimize this threat, we stratified analyses based the timing of drug product monograph donation relative to the number of approved manufacturers for the drug. Third, information regarding the association between the number of generic competitors and drug pricing was based on published analysis which examined only employer-sponsored drug market and may not be representative of the entire U.S. drug market. Fourth, reasons for additional delays in generic market entry as such litigation and the 180-days market exclusivity provisions afforded to the first generic entrant are not universally applicable across products nor are the affected drugs identifiable in the study data. For example, some manufacturers may forfeit the 180 days of exclusivity or may share the claim with other manufacturers. To better isolate the association between of USP drug product monographs and the timing of generic manufacturers’ entry to the market, we focused the analysis on the timing of the third generic entrant and performed secondary analysis evaluating the timing of the second entrant. Finally, estimates of cost savings attributable to drug product monographs were based only on the products examined and focused on a specific calendar year; we did not quantify changes in drug volume due to modeled price effects.

## Conclusion

The high and rising costs of prescription drugs in the United States remains a topic of great concern and public interest. Although there are no simple solutions, one strategy to address these costs is to enhance competition in the generic marketplace. Our findings suggest that pharmacopeial standards, by providing information that facilitates generic manufacturing, may play an important role in this process. While a high proportion of expensive products in the market are currently single-source due to patents or market exclusivity, others represent settings where pharmacopeial standards may help promote more robust generic competition, exert downward pressure on prices, and ultimately, produce considerable societal cost savings.
